# Anti-*Helicobacter pylori* Activity and Gastroprotective Effects of Diacetylcurcumin and Four Metal Derivatives

**DOI:** 10.3390/molecules30193849

**Published:** 2025-09-23

**Authors:** Almanelly Agabo-Martínez, Erika Gomez-Chang, Erick Hernández-Hipólito, Elizabet Estrada-Muñiz, Carolina Escobedo-Martínez, Marco A. Obregón-Mendoza, Raúl G. Enríquez, Libia Vega, Irma Romero

**Affiliations:** 1 Departamento de Bioquímica, Facultad de Medicina, Universidad Nacional Autónoma de México (UNAM), Ciudad Universitaria, Ciudad de México 04510, Mexico; amagcmtz94@gmail.com (A.A.-M.); ergoc@bq.unam.mx (E.G.-C.); erick.hipolito27@gmail.com (E.H.-H.); 2 Departamento de Toxicología, Centro de Investigación y de Estudios Avanzados del Instituto Politécnico Nacional, Zacatenco, Ciudad de México 07360, Mexico; eestrada@cinvestav.mx (E.E.-M.); lvega@cinvestav.mx (L.V.); 3 Departamento de Farmacia, División de Ciencias Naturales y Exactas, Universidad de Guanajuato, Campus Guanajuato, Guanajuato 36050, Mexico; c.escobedo@ugto.mx; 4 Instituto de Química, Universidad Nacional Autónoma de México (UNAM), Ciudad Universitaria, Ciudad de México 04510, Mexico; obregonmendoza@yahoo.com.mx (M.A.O.-M.); enriquezhabib@gmail.com (R.G.E.)

**Keywords:** *Helicobacter pylori*, diacetylcurcumin complexes, gastroprotection, MIC, urease, subacute toxicity

## Abstract

*Helicobacter pylori* is the main etiological factor of gastritis, peptic ulcers, and gastric cancer. This bacterium’s antibiotic resistance has led to a lower eradication rate; therefore, new drugs with anti-*H. pylori* activity are needed. Curcumin exhibits multiple biological activities, but it has low stability and poor bioavailability. To overcome these disadvantages, different metal complexes have been synthesized. The objective of this study was to determine the in vitro anti-*H. pylori* activity of diacetylcurcumin (DAC), DAC_2_-Cu, DAC_2_-Zn, DAC_2_-Mn, and DAC_2_-Mg by obtaining the minimum inhibitory concentration of bacterial growth, and to investigate some mechanisms by which they could affect the bacteria (urease and DNA gyrase activities). Moreover, their gastroprotective potential was assayed in an ethanol-induced gastric ulcer model in mice. The results showed that DAC_2_-Cu and DAC_2_-Zn have good anti-*H. pylori* activity, exhibit specific activity against this bacterium, inhibit the urease activity, and provide 70% gastroprotection at a dose of 200 mg/kg of body weight. In a subacute toxicity study in mice, DAC_2_-Cu and DAC_2_-Zn did not cause death or any deleterious symptoms, nor did they have a significant effect on serum and urine biochemical parameters compared to control mice. These compounds are promising candidates for use in *H. pylori* eradication schemes.

## 1. Introduction

*Helicobacter pylori* is a gastric human pathogen that infects 44.3% of the world’s population [1]. Eighty percent of infected people are asymptomatic; however, all of them have gastritis, 10–20% develop duodenal or gastric ulcer, 1–2% progress toward gastric cancer, and less than 1% evolve into MALT-type lymphoma [2]. The outcome of the infection depends on three key elements: the bacterial genotype, the host’s genetic predisposition, and environmental factors such as diet, among others.

This bacterium has developed several pathogenic factors that allow it to survive, cause damage to the gastric epithelium, and persist in the stomach, despite the hostile acidic environment. The eradication of *H. pylori* is crucial for reducing gastric mucosal inflammation, promoting ulcer healing, lowering the risk of gastric cancer, and decreasing mortality associated with these conditions. Currently, there is no way to prevent *H. pylori* infection except for basic hygiene rules.

Standard triple therapy is the most common treatment for *H. pylori* eradication, which is based on the administration of a proton pump inhibitor (PPI) in conjunction with two antibiotics, usually clarithromycin and amoxicillin or metronidazole or tetracycline for 14 days. However, this therapy often fails, so quadruple therapy is increasingly used as a first-line treatment. In addition to PPI and antibiotics, this regimen includes bismuth salts. Other antibiotics used may comprise quinolones, such as levofloxacin [3]. The main reasons for treatment failure are escalating antibiotic resistance and adverse effects that make patients uncomfortable and lead them to abandon therapy [4]. Therefore, it is important to explore novel, more efficacious, and less aggressive treatments for *H*. *pylori* eradication.

**Figure 1:**
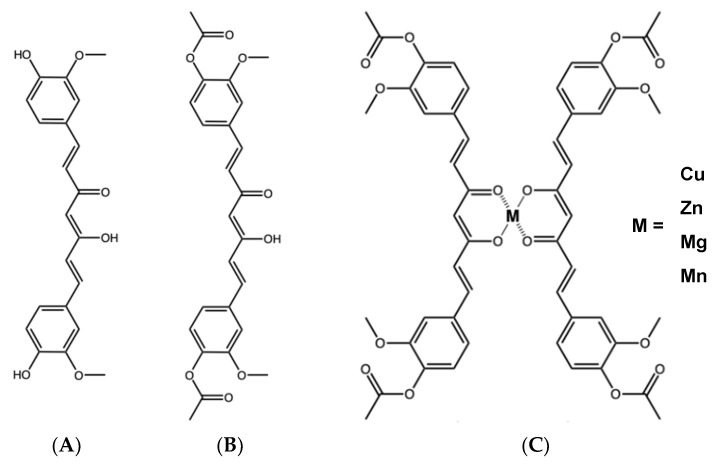
Chemical structure of (**A**) curcumin, (**B**) diacetylcurcumin, and (**C**) its metallic complexes.

Curcumin (C_21_H_20_O_6_) is a compound obtained from the rhizome of *Curcuma longa*, which is used in Asia as a food flavoring, coloring, food preservative, and even in other countries as a dietary supplement [5]. This compound has three chemical entities in its structure: two aromatic rings with o-methoxylated phenolic groups connected through a 7-carbon bond with a β-diketone α, β-unsaturated center (Figure 1A), which are responsible for its multiple biological activities [6]. Many in vitro and in vivo studies have demonstrated its antioxidant, antiproliferative, anti-inflammatory, anticancer, and antimicrobial activities [7].

The activity of curcumin against *H. pylori* has been assessed in vitro, revealing a Minimum Inhibitory Concentration (MIC) ranging from 5 to 50 μg/mL against clinical isolates of the bacterium [8,9]. An in vivo study demonstrated the therapeutic potential of curcumin in a murine model of *H. pylori* infection, where it effectively eradicated the bacteria and ameliorated gastric damage induced by the infection [9]. Additional studies found that curcumin reduced the activation of proinflammatory mediators [10]. Notably, this compound has demonstrated non-toxicity in humans at doses up to 12 g per day [11].

In contrast to the promising activity demonstrated by curcumin in preclinical in vitro and in vivo studies, clinical trials have revealed that curcumin shows a poor eradication effect against *H. pylori* [12]. This limitation can be attributed to the compound’s low bioavailability in the body. To address this challenge and enhance the pharmacokinetic profile of curcumin, nanoparticles, micelles, and synthetic derivatives have been developed [13]. A notable advancement in this area involves leveraging the chelating properties of curcumin’s β-diketone center to synthesize stable metal complexes [6]. Diacetylcurcumin (DAC) (Figure 1B) is a derivative of curcumin in which the phenolic OH groups have been acetylated. Dr. Enriquez’s group successfully synthesized four metal complexes from DAC with a 2:1 DAC/metal ratio (Figure 1C): DAC_2_-Cu, DAC_2_-Zn, DAC_2_-Mn, and DAC_2_-Mg [14]. These compounds exhibited antioxidant activity in a lipid peroxidation model and cytotoxic effects against three cancer cell lines: colorectal adenocarcinoma (HCT-15), breast adenocarcinoma (MCF-7), and lung adenocarcinoma (SKLU-1), and no acute toxicity in a murine model (LD > 3 g/kg) [14].

Based on the above-described effects of curcumin on *H. pylori* and its associated diseases, it would be desirable to find out whether DAC and its metal derivatives retain these properties. Therefore, the present work aimed to determine the effect of DAC, DAC_2_-Cu, DAC_2_-Zn, DAC_2_-Mn and DAC_2_-Mg on the growth of *H. pylori* and their impact on two important enzymes of bacterial metabolism such as urease, an enzyme essential for colonization and survival in the acid environment of the stomach [15], and DNA gyrase, a key enzyme in DNA metabolism [16]. We also assessed the anti-ulcerogenic effect of the compounds. This information provides an essential basis for the use of these curcumin derivatives in anti-*H. pylori* therapies.

## 2. Results

### 2.1. Anti-Helicobacter pylori Activity

Initially, we evaluated the anti-*H. pylori* effect of curcumin, DAC, and their metallic complexes. All the tested compounds showed inhibitory activity against *H. pylori* growth, considering the MIC values obtained (Table 1). This activity was higher than the inhibition of metronidazole, but none surpassed the MIC of clarithromycin. DAC_2_-Cu and DAC_2_-Mn had the lowest MIC, followed by DAC_2_-Mg and DAC_2_-Zn.

#### Activity Versus Other Bacteria

To investigate if the inhibitory activity of the compounds was specific to *H. pylori*, their effect on other bacteria was tested [*Escherichia coli* (EPEC), *Salmonella enterica* serovar Typhimurium, and *Staphylococcus aureus*]. Experiments were performed up to a concentration of 62.5 µg/mL, equivalent to the MIC for DAC. At this concentration, none of the compounds completely inhibited the growth of any of the bacteria tested (Table 2); DAC and its metal derivatives inhibited less than 25%. Curcumin, which was used as a control, inhibited in a higher percentage; however, these values are lower than the effect of curcumin against *H. pylori* (100% inhibition at 1.95 µg/mL).

### 2.2. Helicobacter pylori Urease Inhibition

**Figure 2:**
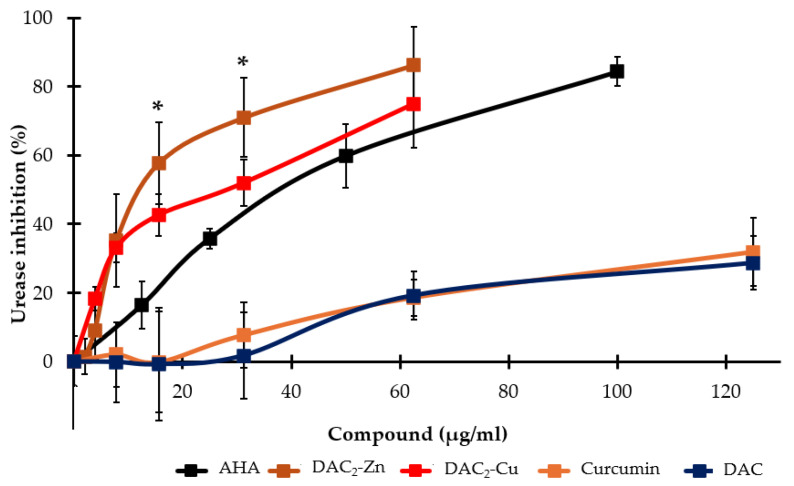
Effect of DAC and its metal derivatives on *H. pylori* urease activity. AHA: Acetohydroxamic acid, DAC: diacetylcurcumin. Data presented as mean ± SD. *n* = 3 per value. * Statistically significant difference between DAC_2_-Cu and DAC_2_-Zn (*p* < 0.05).

*H. pylori* urease is an enzyme that is crucial for the colonization, survival, and pathogenicity of the bacterium. Figure 2 shows that DAC_2_-Zn inhibits 86.3% of urease activity at 62.5 µg/mL, while DAC_2_-Cu inhibits 75% (there was no statistically significant difference between both at this concentration (*p* > 0.05)). The respective IC_50_ values calculated were 13.0 and 21.2 µg/mL (12.4 and 20.4 µM). These compounds had better activity than the positive control, acetohydroxamic acid (AHA), which has an IC_50_ value of 35.7 μg/mL (488.4 µM). Curcumin and DAC inhibited 30% of urease activity at 125.0 μg/mL (339.3 and 276.2 µM, respectively).

In contrast to the inhibition of urease activity, DAC_2_-Mn exhibited a dose-dependent activation, reaching a maximum of 76.3% at 62.5 μg/mL (56.1 μM). Meanwhile, DAC_2_-Mg did not affect the enzyme.

### 2.3. Effect on DNA Gyrase Activity

DNA gyrase is another essential enzyme for *H. pylori*. Its function is critical for DNA replication, recombination, and transcription. Except for DAC_2_-Mn, none of the compounds had a significant effect on *E. coli* DNA gyrase supercoiling activity. DAC_2_-Mn inhibited gyrase activity by 40% at 55.7 μg/mL (50 µM) (Table S1).

### 2.4. Gastroprotective Effect In Vivo

**Figure 3:**
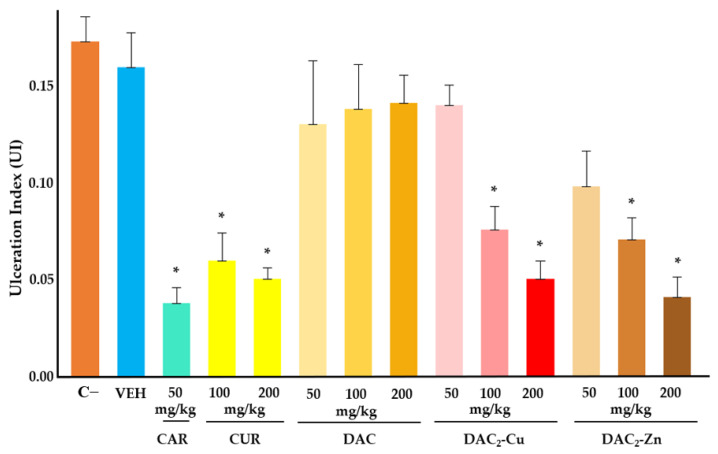
The anti-ulcerogenic effects of DAC, DAC_2_-Cu, and DAC_2_-Zn in an acute ethanol-induced gastric ulcer model. C−: Negative control [Isotonic sodium saline solution (NSS)], VEH: Vehicle [sodium carboxymethylcellulose (CMC) 0.5%], CAR: Carbenoxolone. CUR: Curcumin. DAC: Diacetylcurcumin. *n* = 6 per treatment. Data presented as mean ± SEM. * Statistically significant difference with respect to negative control (*p* < 0.05).

After demonstrating the anti-*H. pylori* activity of the studied compounds, their gastroprotective activity was evaluated using an acute ethanol-induced gastric ulcer model in mice. It was observed that the DAC_2_-Cu and DAC_2_-Zn were the only two compounds that significantly reduced the ulcer index in a dose-dependent manner (Figure 3). At a concentration of 200 mg/kg body weight (BW), both compounds exhibited approximately 70% gastroprotective activity (Table 3), which was statistically equivalent to the activity of curcumin at the same concentration and carbenoxolone at 50 mg/kg BW. DAC, DAC_2_-Mn, and DAC_2_-Mg did not have statistically significant gastroprotection (Table 3).

**Figure 4:**
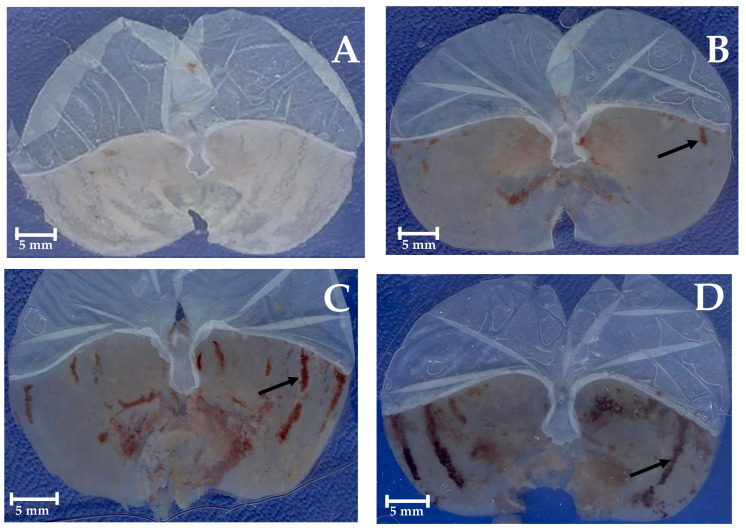

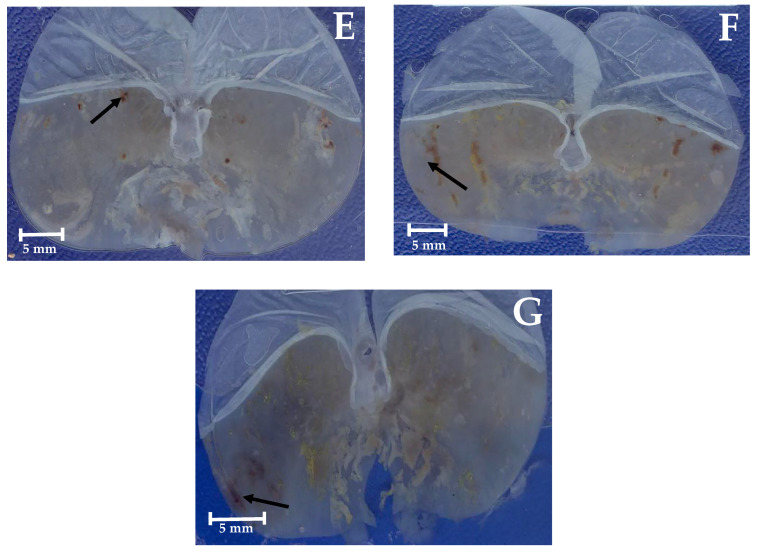
Representative stomachs. (**A**) Normal stomach. (**B**) Carbenoxolone (50 mg/kg BW). (**C**) Negative control, isotonic sodium saline solution (NSS). (**D**) Vehicle, 0.5% CMC. (**E**) Curcumin (200 mg/kg BW). (**F**) DAC_2_-Cu (200 mg/kg BW). (**G**) DAC_2_-Zn (200 mg/kg BW). Hemorrhagic ulcers (black arrows).

Macroscopic images of representative stomachs are shown in Figure 4. In contrast to a normal stomach (Figure 4A), the negative control and the vehicle stomachs (Figure 4C and Figure 4D) presented severe hemorrhagic ulcer areas in the glandular epithelium (black arrows), while those treated with carbenoxolone, positive control (50 mg/kg BW) (Figure 4B), curcumin, DAC_2_-Cu, and DAC_2_-Zn (Figure 4E, Figure 4F and Figure 4G, respectively) with 200 mg/kg BW, exhibit few visible lesions on the stomach surface.

**Figure 5:**
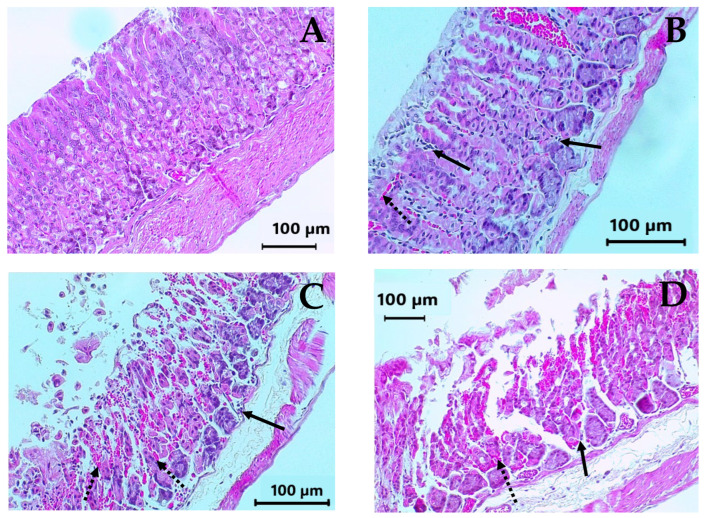

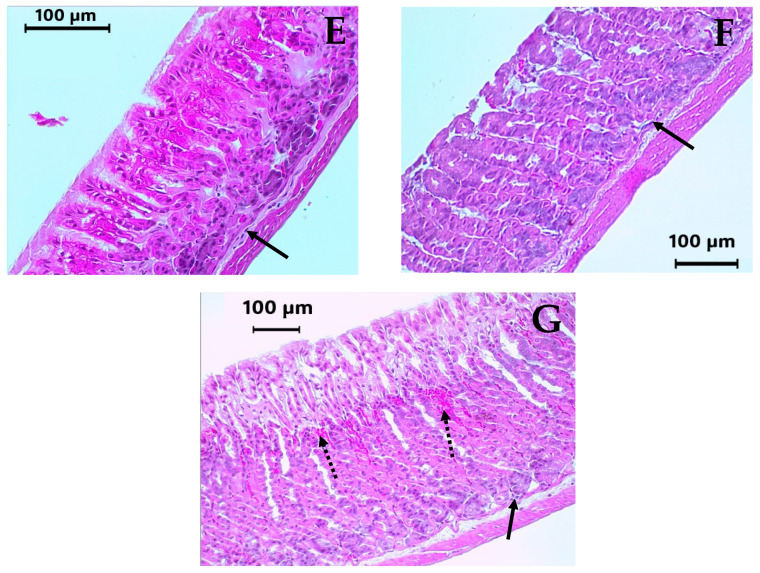
Representative microphotographs of the glandular portion of the mouse stomach after administration of different treatments. (H&E, 200×). (**A**) Normal stomach. (**B**) Carbenoxolone (50 mg/kg BW). (**C**) Negative control, NSS. (**D**) Vehicle, 0.5%. CMC (**E**) Curcumin (200 mg/kg BW). (**F**) DAC_2_-Cu (200 mg/kg BW). (**G**) DAC_2_-Zn (200 mg/kg BW). Dotted black arrows: erythrocyte infiltrate. Black arrows: inflammatory infiltrate.

The histological examination of the stomachs is shown in Figure 5. The lining of the normal stomach shows a healthy epithelium, with normal glandular structures, and virtually no inflammatory cells (Figure 5A). This structure is similar to that observed in the stomach treated with 50 mg/kg BW of carbenoxolone. Although this compound is a known gastroprotective agent that favors the gastric glands’ preservation, certain damage, such as mild edema in the submucosa, as well as some inflammatory and erythrocyte infiltrates, can be observed (Figure 5B). In the negative control and vehicle representative stomachs, the presence of abundant erythrocytes and inflammatory cell infiltrates in the mucosa are evident. Also, there is loss of the glandular architecture, exhibited by destruction of the epithelial cells, as well as edema in the submucosa (Figure 5C and Figure 5D). With the highest tested dose of DAC_2_-Cu and DAC_2_-Zn (200 mg/kg body weight), maximum gastroprotection was achieved, as evidenced by the preservation of the glandular mucosa structure and the reduction in inflammatory and erythrocytic infiltrates (Figure 5F and Figure 5G). These results were also obtained when using curcumin as treatment (200 mg/kg BW); however, low mucosa damage persisted, which could be seen by some red cell blood infiltration (Figure 5E).

### 2.5. Subacute Toxicity Assay of DAC_2_-Cu and DAC_2_-Zn

Subacute toxicity was evaluated by administering a single daily oral dose of 200 mg/kg BW of either DAC_2_-Cu or DAC_2_-Zn to groups of CD1 mice for 28 days, in addition to a control group that received the vehicle (0.5% carboxymethyl cellulose solution, CMC). Recovery groups were also included for the same variables, which were observed for 28 days after the treatment was suspended.

**Figure 6:**
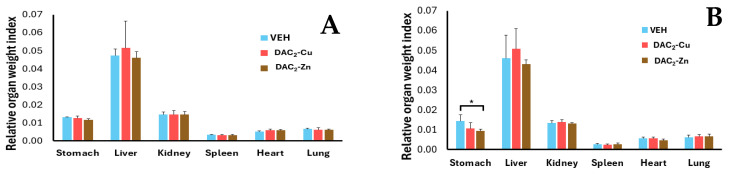
Relative organ weight index of mice. (**A**) experimental phase, and (**B**) recovery phase. There were no statistically significant differences (*p* < 0.05) in the relative weight of the organs between the control (VEH) and the DAC_2_-Cu and DAC_2_-Zn treatments, except in the case of the stomachs of the mice in the recovery group that received DAC_2_-Zn, which were significantly smaller than their controls (*p* < 0.05) (*). Data presented as mean ± SD.

No deaths were recorded in any of the groups, nor were any changes in the general behavior of the animals, except mild fur changes in all groups during the 56 days of the experiment, not attributed to the treatments. There were no significant differences (*p* > 0.05) in body weight between the treatment groups and the control group throughout the experiment (Figure S1). In general, the organ-weight ratio was also similar between the control and treatment groups for both the experimental and recovery phases (Figure 6). It was found that the stomachs of animals treated with DAC_2_-Zn, and after the recovery stage, were significantly smaller (*p* = 0.031) than their corresponding experimental group. Similarly, the spleens of control mice (VEH) in the recovery phase are smaller (*p* = 0.040) than their experimental counterparts.

Macroscopic examination of the organs revealed no treatment-related findings in the general appearance and the architecture of the vital organs, except for a darkening in the upper right lobe of the lung in 3/5 animals of the DAC_2_-Zn experimental group and 2/4 animals of the DAC_2_-Cu recovery group (Table S2 and Figure S2).

**Figure 7:**
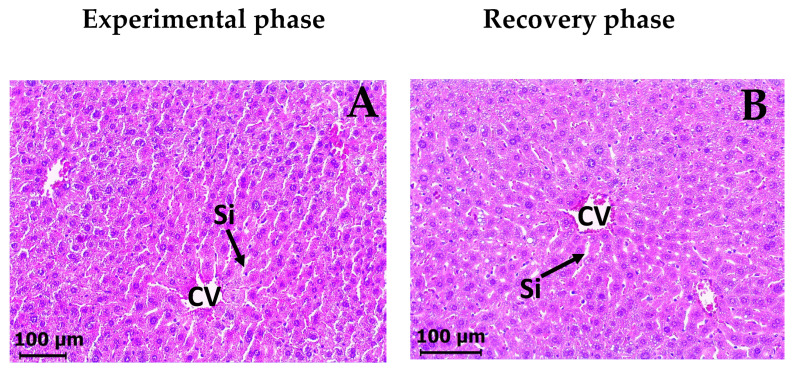

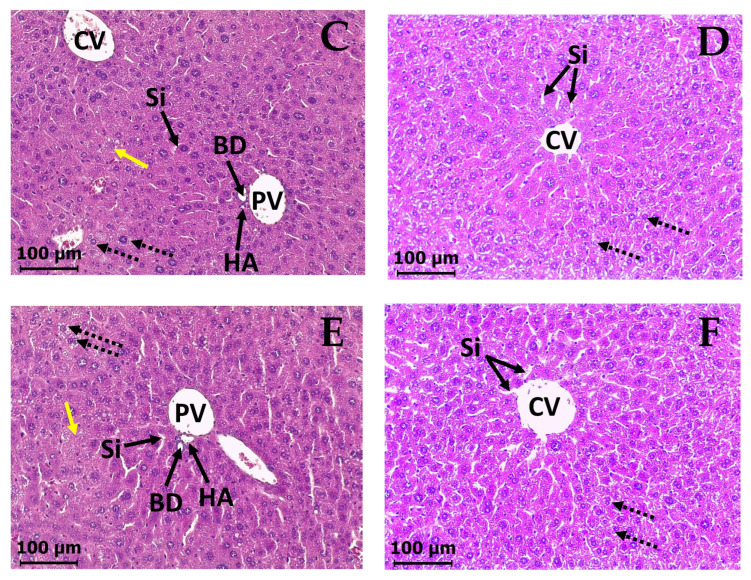
Representative microphotographs of a mouse liver section after DAC_2_-Cu and DAC_2_-Zn administration. (H&E, 200). (**A**) Control, vehicle 0.5% CMC, (**C**) DAC_2_-Cu, 200 mg/kg BW, and (**E**) DAC_2_-Zn, 200 mg/kg BW of the experimental phase. (**B**) Control. (**D**) DAC_2_-Cu, and (**F**) DAC_2_-Zn, corresponding images from the recovery phase. (PV) portal vein. (Si) sinusoids. (HA) hepatic artery. (BD) bile duct. (CV) central vein. Vacuolar degeneration (dotted black arrows). Apoptotic cells (yellow arrows).

In the histopathological examination, the livers of VEH groups (experimental and recovery phases) stained with H&E exhibited typical histological characteristics (Figure 7A and Figure 7B). They had a normal structure with central veins and portal triads. The hepatocytes were arranged in cords that radiated outward from the central veins (plates), and there was a network of sinusoids between the hepatocyte cords. The hepatocytes showed a typical feature in the shape and size, with eosinophilic cytoplasm containing small vacuoles and visible acidophilic nuclei. In contrast, the liver of DAC_2_-Cu and DAC_2_-Zn treated groups exhibited a disorganized cellular structure, with loss of the radial liver plate and sinusoids, and extensive central veins congestion was observed. The hepatocytes showed slightly acidic cytoplasm and increased vacuolization, compared to the control group (Figure 7C and Figure 7E). Some cells in degeneration/necrosis can also be seen (Figure S3). However, the histological images of the recovery phase for both treatments showed a clear improvement of damaged liver tissue (Figure 7C vs. Figure 7D and Figure 7E vs. Figure 7F), and the radial organization of the hepatocyte cords and sinusoids is restored. Likewise, congestion of the central veins is reduced. The acidophilic cytoplasm of the hepatocytes disappears completely, although vacuolization persisted.

**Figure 8:**
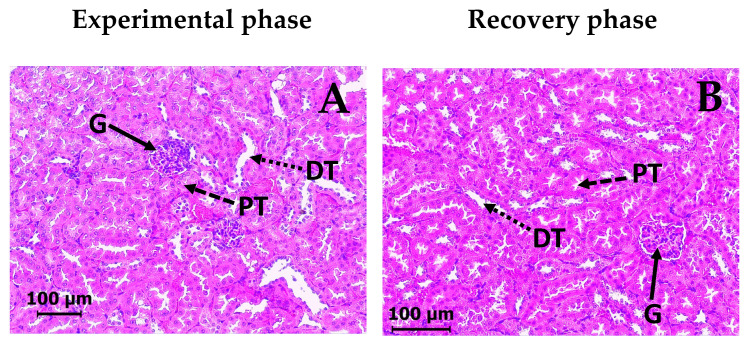

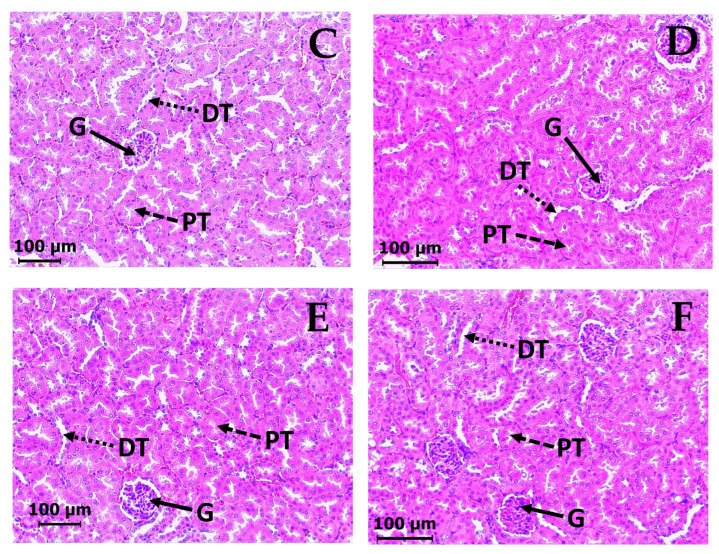
Representative microphotographs of a mouse kidney section after DAC_2_-Cu and DAC_2_-Zn administration. (H&E, 200×). (**A**) Control, vehicle 0.5% CMC, (**C**) DAC_2_-Cu, 200 mg/kg BW, and (**E**) DAC_2_-Zn, 200 mg/kg BW of the experimental phase. (**B**) Control. (**D**) DAC_2_-Cu, and (**F**) DAC_2_-Zn, corresponding images from the recovery phase. (G) glomerulus. (PT) proximal tubule. (DT) distal tubule.

Regarding the kidney, the control VEH group of mice showed normal histological structure of the renal parenchyma, with intact tubular lining cells and glomeruli as evidenced by H&E staining (Figure 8A). The groups treated with DAC_2_-Cu and DAC_2_-Zn showed no histopathological alterations (Figure 8C and Figure 8E). Similarly, all recovery groups demonstrated normal structural organization (Figure 8B, Figure 8D and Figure 8F).

The clinical biochemistry results are presented in Table 4. No statistically significant changes were observed in DAC_2_-Cu and DAC_2_-Zn treated animal groups compared to the controls in any of the serum parameters tested: total protein, albumin, creatinine, total bilirubin, glucose, and alanine aminotransferase and lactate dehydrogenase enzymes in either the experimental or recovery phases. The same can be observed for total protein, creatinine, and glucose in urine samples.

## 3. Discussion

Several studies have recently focused on the identification of novel alternatives for *H. pylori* eradication that exhibit superior efficacy in comparison with conventional therapeutic regimens, reduced adverse effects, and greater accessibility for patients. Curcumin, extracted from the rhizome of *Curcuma longa*, has attracted significant attention due to its numerous reported pharmacological effects [17]. These effects include anti-*H. pylori* activity, antiulcerogenic properties, and anticancer activity [9,18,19,20]. However, the limited bioavailability of curcumin due to high decomposition, low absorption, and rapid metabolism and excretion has limited its therapeutic use [17]. Intending to enhance the pharmacokinetic profile of the compound, Dr. Enriquez’s group used DAC to synthesize DAC_2_-Mg, DAC_2_-Mn, DAC_2_-Cu, and DAC_2_-Zn [14]. In this context, we decided to explore whether the chemically modified compounds could be exploited for use as new alternatives for *H. pylori* eradication or even for the treatment of its associated diseases, such as gastric ulcers.

DAC and its derivatives successfully inhibited the growth of *H. pylori* (Table 1). The activity of all compounds was higher than metronidazole’s effect, though not compared to clarithromycin. Since the *H. pylori* strain used here is metronidazole-resistant, having compounds that improve the antimicrobial activity could contribute to eradication therapies. DAC_2_-Cu and DAC_2_-Mn have the best anti-*H. pylori* activity (MIC = 15.6 μg/mL) among DAC and its metal derivatives.

The anti-*H. pylori* activity of natural plant-derived products has previously been reported. These products include flavonoids, quinones, coumarins, terpenoids, alkaloids, and phenolic compounds, such as curcumin, whose MICs range from 0.156 to 25 μg/mL, or greater than 100 μg/mL for some coumarins [21].

Curcumin was included in the assay as a control, exhibiting an MIC of 1.95 µg/mL. This value is lower than those previously reported [8,9]; the difference could be due to the method used for the evaluation, which in this work was by broth dilution, and in the other cases were by agar dilution and agar well diffusion.

Interestingly, neither DAC nor the derivatives improved the MIC of curcumin. This finding suggests that acetylation of the molecule is deleterious to anti-*H. pylori* activity; however, the formation of metal complexes, particularly with copper and manganese, enhanced the DAC effect (Table 1).

To explore the specificity of the compounds, their effect against other bacteria was investigated. At the highest concentration used (62.5 µg/mL), at which DAC and all its metal complexes inhibited *H. pylori* at 100%, none of them significantly inhibited the growth of *E. coli* (EPEC), *Salmonella enterica* serovar Typhimurium, and *Staphylococcus aureus* (Table 2). These compounds do not appear to act based on the type of bacterial cell wall, as *H. pylori,* EPEC, and *S. enterica* are Gram-negative, whereas *S. aureus* is Gram-positive. This indicates a specificity toward *H. pylori*, which could be advantageous in eradication therapies by providing a more precise and effective antibiotic with a lower risk of antimicrobial resistance development. However, this should be explored with a larger number of pathogenic bacteria.

This assay also included curcumin as a control, this compound is known to have a broad in vitro bactericidal spectrum against Gram-positive and Gram-negative bacteria, including members of the species tested in this work [22,23]. At the concentrations used in this study, curcumin inhibits, but not completely, the growth of EPEC, *S. enterica*, or *S. aureus*. However, compared to the effects of DAC and its metal derivatives, curcumin is more efficient. Although there are no data on the antibacterial activity of DAC metal complexes, there are few reports on the effect of DAC on the growth of certain bacteria. Tajbakhsh et al. [24] found that the compound did not affect *S. aureus*, *S. epidermidis*, *Pseudomonas aeruginosa*, or *E. coli* at concentrations up to 373 μg/mL, which is consistent with our results. On the other hand, a mild effect against *Mycobacterium tuberculosis* was observed (MIC = 200 μg/mL) [25]. Also, a significant DAC activity was reported against *Enterococcus faecalis* [26] and *Streptococcus mutans* [27], with an MIC value of 15.6 μg/mL for both.

In an effort to elucidate the mechanism by which DAC and the metal complexes inhibit *H. pylori*, their effect on the activity of two essential enzymes, urease and bacterial gyrase, was evaluated.

The urease of *H. pylori* is vital for colonization in the human stomach. This cytosolic enzyme, which represents 6–10% of the total protein, uses nickel as a cofactor and is primarily responsible for the bacterium’s acid acclimation. The hydrolysis of urea into ammonia and carbon dioxide contributes to buffering the environment of the bacteria by protecting them from damage caused by gastric acid and allows maintenance of proton motive force for bacterial growth in the gastric environment [28].

Other functions attributed to *H. pylori* urease include reducing mucus viscosity, altering epithelial integrity, inducing proinflammatory cytokines, and protecting against reactive oxygen species. Urease activity is also associated with an increased risk of histopathological alterations and gastric carcinogenesis [15].

It has been reported that *H. pylori* strains that do not express urease are not able to colonize the human stomach [29,30,31]. Considering the role of this enzyme in the colonization and virulence of the bacterium, it has become an important therapeutic target.

Figure 2 shows that DAC_2_-Zn and DAC_2_-Cu were the curcuminoids that most effectively inhibited *H. pylori* urease activity in a concentration-dependent manner. They reached 86.3% and 75% inhibition, respectively, at 62.5 μg/mL. Higher concentrations could not be used due to solubility issues. Analyzing the IC_50_ values obtained for these complexes (12.4 µM for DAC_2_-Zn and 20.4 µM for DAC_2_-Cu) and the value calculated for AHA (a competitive and irreversible inhibitor of the enzyme with an IC_50_ value of 488.4 µM) reveals the higher efficiency of the metal complexes. Curcumin and DAC, on the other hand, inhibited urease by less than 30% at 125 μg/mL (339.3 and 276.2 µM, respectively). Considering that these two compounds retain a β-diketonic center in their structure, which has transition metal chelating capacity (e.g., nickel), one might expect these two compounds to inhibit urease; however, this was not observed. Unexpectedly, DAC_2_-Mn increased urease activity by 76.3% at 62.5 µg/mL. This is not particularly relevant to this study, but urease activation could be useful in other applications, such as in the bioremediation industry [32]. One possible explanation for the activating effect of DAC_2_-Mn could be its interaction with the protein in such a way that it modifies its structure, making catalysis at the active site more efficient; further studies, such as molecular docking, are required to establish whether this possibility is feasible.

Comparing the *H. pylori* growth inhibitory effects of DAC_2_-Zn and DAC_2_-Cu (MIC = 31.2 and 15.6 µg/mL, respectively) with their corresponding urease enzyme inhibition (~80% at 62.5 µg/mL, in both cases), more compound is required to inhibit the enzyme than to stop bacterial growth. DAC_2_-Mg did not affect urease but had anti-*H. pylori* activity (MIC = 31.2 µg/mL), while DAC_2_-Mn activated the enzyme, which could be advantageous for *H. pylori*, but in contrast, it was one of the compounds with higher activity against the bacteria (MIC = 15.6 µg/mL). These data lead us to conclude that the anti-*H. pylori* mechanism of action of DAC and its metal derivatives is not through urease inhibition, although it could be a secondary target for DAC_2_-Zn and DAC_2_-Cu.

Another essential enzyme of *H. pylori* is DNA gyrase, a type II topoisomerase found in bacteria but not in mammals. This enzyme is unique in introducing ATP-dependent negative supercoils during DNA replication and transcription, preventing strand stress [16]. Quinolones are the only antibiotics used in *H. pylori* eradication therapies that inhibit DNA gyrase activity, leading to DNA fragmentation and bacterial death [16,33]. However, the emergence of *H. pylori* strains resistant to levofloxacin, the quinolone most used in traditional therapies, has made DNA gyrase an important pharmacological target that has been studied in recent years.

In this work, the *E. coli* DNA Gyrase Drug Screening kit from TopoGEN was used to determine the effect of DAC and its metal derivatives on the supercoiling activity of the enzyme. DNA gyrases are highly conserved enzymes in function and structure across bacteria [34,35]; therefore, the results obtained are likely to reflect the effects that the compounds could have on the *H. pylori* enzyme.

Under the experimental conditions and within the concentration range of 0.1–50 μM, none of the compounds, including curcumin, inhibited DNA gyrase activity. On the contrary, the positive control ciprofloxacin inhibited 100% the supercoiling activity with 3 µM. This suggests that bacterial DNA gyrase is unlikely to be the target of DAC or its metal derivatives in *H. pylori.* However, this result on *H. pylori* DNA gyrase remains to be corroborated.

To further explore the pharmacological properties of the compounds, the gastroprotective effect of DAC and its metal derivatives was investigated in a model of acute ulceration induced with absolute ethanol in CD1 mice. This model is widely used to study the efficacy of compounds that are believed to have gastroprotective effects.

Ulcers are lesions that originate in the gastric mucosa due to an imbalance between protective and harmful mucosal factors. Ethanol causes acute hemorrhages, vascular congestion, edema, and necrosis of the mucosa by solubilization of the mucus layer, which leaves the superficial mucous cells in contact with stomach acid and proteolytic enzymes that damage the cell membrane. Exposure to ethanol also increases HCl secretion, decreases blood flow, increases permeability and free radical production, and alters bicarbonate secretion, all these factors together alter the structure of gastric mucosa [36].

Among the compounds tested, only DAC_2_-Cu and DAC_2_-Zn showed gastroprotective activity, with maximum values of 71.2% and 76.6%, respectively, at 200 mg/kg BW (Table 3). Macroscopic and microscopic histological analyses of the stomachs confirmed the anti-ulcer effect of the compounds. Their gastroprotective activity was comparable to the positive control, carbenoxolone, which showed 78.3% protection at 50 mg/kg BW.

Similarly, the gastroprotective effect of the compounds was comparable to the effect obtained with 200 mg/kg of curcumin (71.1%). Curcumin’s antiulcer activity has been widely demonstrated in many ulceration models [37,38,39,40]. Percentages vary depending on the agent used to generate the ulcer, but differences have also been reported in the same model. For instance, using an ethanol-induced ulcer model in rats similar to the one in this study, Chattopadhyay et al. [37] reported 95% gastroprotection at a dose of 25 mg/kg, whereas Czekaj et al. [39] obtained 89% at 100 mg/kg.

The differences in effects observed in in vitro compound susceptibility experiments and in vivo gastroprotection experiments are due to the different metabolic conditions to which the complexes are exposed. In vitro experiments involve direct contact between the compounds and the bacteria, whereas gastroprotection experiments may involve a local effect in the stomach or exposure to the animal’s own biotransformation.

The present work is the first report on the antiulcer activity of DAC and its metal complexes. To our knowledge and regarding complexes with curcumin, the gastroprotective activity of a Zn(II)–curcumin complex in solid dispersions (SDs) has been reported in pylorus-ligature [41], cold-restraint stress [42], and ethanol [43] induction of gastric ulcer models. The results showed gastroprotective percentages of 73.7, 81.1, and 75.0%, respectively, at a dose of 48 mg/kg of BW. The authors also report that the percentage achieved with the complex is slightly higher than the gastroprotective effect of curcumin SDs. The complex was characterized using mass spectrometry, thermal gravimetry, and differential thermal analysis. These analyses indicated that the curcumin-Zn(II) complex contains a zinc atom that is coordinated through the keto-enol group of curcumin, as well as an acetate group and a water molecule [41].

Although our results confirm the anti-ulcerogenic effect of DAC_2_-Cu and DAC_2_-Zn, further studies are needed to elucidate their precise gastroprotective mechanism.

The in vitro and in vivo results obtained in this study lead us to identify two of the compounds studied, DAC_2_-Cu and DAC_2_-Zn, as candidates for use in *H. pylori* eradication therapies and in the treatment of diseases associated with it. Both compounds have very good inhibitory activity against *H. pylori* growth (MIC = 15.2 and 31.2 μg/mL, respectively), which appears to be species-specific since they did not inhibit other bacteria; they inhibit the urease of the bacterium (IC_50_ = 13 and 21.2 μg/mL, respectively), which may be one of their mechanisms of action; and they also have a good gastroprotective effect (71% and 76%, respectively, with 200 mg/kg). Therefore, in order to be implemented in *H. pylori* eradication therapies and in the treatment of associated diseases, such as peptic ulcers, it is necessary to determine their pharmacological biosafety. Toxicity studies provide essential data on a compound’s safety profile, identifying risks before human clinical trials.

In this regard, the only study conducted to evaluate the in vivo toxicity of DAC and its four metal derivatives was an acute toxicity study in a murine model, conducted by Meza-Morales et al. [14]. The authors observed no clinical signs of toxicity with any of the compounds administered orally at doses up to 3000 mg/kg BW. Slight hair frizzing was only observed with DAC_2_-Cu and DAC_2_-Mn treatments. However, these results only provide an initial indication of the gross signs of toxicity of the compounds and do not reflect long-term continuous exposure.

Eradication therapy for *H. pylori* can vary from 10 to 14 days, depending on the type, to achieve the best effectiveness rates [3]. Therefore, in accordance with OECD Guidelines for the Testing of Chemicals 407 [44], we consider that a 28-day study at a fixed dose of 200 mg/kg BW would provide the basic necessary information on the effects of repeated oral exposure to DAC_2_-Cu and DAC_2_-Zn. The dosage was chosen based on our previous in vitro and in vivo results.

The subacute toxicity study was divided into two phases. In the first experimental phase, one group was treated with the vehicle, and two others were treated with DAC_2_-Cu and DAC_2_-Zn at a dose of 200 mg/kg BW for 28 days. In the second phase, the recovery phase, three other groups were treated the same as in the first phase, but after finishing administration, these groups were observed for an additional 28 days to detect any toxic effects or reversibility of the effects produced by the compounds.

During our study, no mortality was observed in any of the groups during the 56-day evaluation period (28 days of experimental phase and 28 days of recovery phase). The administration of DAC_2_-Cu and DAC_2_-Zn did not cause abnormal alterations in the behavior of mice. No significant modifications in body weight between treated and untreated groups of animals were found. In general, no macroscopic changes in the organs or considerable alterations in the relative organ weight were observed. These findings suggest that the experimental compounds do not alter the normal growth and development of the animals. Furthermore, the biochemical parameters that were determined did not show any significant alterations compared to the control group.

The major sites of metabolism and excretion of many chemicals and drugs are the liver and the kidneys. Histopathological analysis demonstrates no renal toxicity. Tissue samples show no evidence of cellular damage, inflammation, or other abnormalities, suggesting that DAC_2_-Cu and DAC_2_-Zn are not nephrotoxic. On the other hand, the analysis of the liver indicates that these compounds induce some changes in the liver structure (Figure 7C and Figure 7E), such as loss of overall cellular structure, venous congestion, and increased vacuolization of hepatocytes. However, these abnormalities are almost completely reversed 28 days after the administration of the compounds has ended (recovery phase, Figure 7D and Figure 7F), which demonstrates that the injury caused by the compounds is reversible and that the tissue is expected to recover completely. The observed changes in the liver’s histological architecture 28 days after administration of the compounds probably represent the onset of a low-grade, temporary inflammatory process, as there is no massive infiltration of immune cells. In these circumstances, there is no alteration to liver-specific biochemical parameters, suggesting that the organ’s physiology has not yet been affected.

As these compounds are intended for use within a sufficient timeframe for treating *H. pylori*-related diseases (10–14 days), they do not pose a health risk. However, based on the results obtained in this study, prolonged administration is not recommended until any potential long-term effects on liver function have been confirmed or ruled out.

Considering that the lability of the complexes within the organism has not yet been demonstrated, it would be advisable to quantify the concentrations of free ions and ascertain their potential impact on the experiments conducted in this study.

The findings of this study are promising because they provide concrete data on the toxicological safety of the curcuminoid complexes DAC_2_-Cu and DAC_2_-Zn when administered daily for 28 days in an in vivo model. However, to evaluate the safety and therapeutic potential of these compounds in humans, further research is necessary at a preclinical level to understand their mechanism of action, to know more about their toxicological profile, and their therapeutic efficacy. Finally, their pharmacokinetic and pharmacodynamic properties must be assessed.

## 4. Materials and Methods

### 4.1. Curcumin, DAC, and Its Metal Derivatives

Diacetylcurcumin and its metal derivatives (DAC_2_-Cu, DAC_2_-Zn, DAC_2_-Mn, and DAC_2_-Mg) were synthetized by Dr. Raúl G. Enríquez’s group from the Chemistry Institute of UNAM according to reference [14]. Curcumin was extracted from a natural source through conventional procedures and subsequently purified by crystallization.

### 4.2. Strains and Growing Conditions

*Helicobacter pylori ATCC 43504* was cultured on Casman Agar (Becton, Dickinson and Company, Sparks, MD, USA) supplemented with 5% defibrinated sheep blood, 10 mg/L vancomycin, 5 mg/L trimethoprim, 2 mg/L amphotericin B, and 2.5 mg/L polymyxin B at 37 °C for 24 h under microaerophilic conditions (10% CO_2_, 5% O_2_, 85% N_2_). Cultures were stored at −70 °C in Brucella broth (Becton, Dickinson and Company, Sparks, MD, USA) supplemented with 10% fetal bovine serum (Gibco BRL Life Technologies Corporation, Grand Island, NY, USA) and 10% glycerol. The strains were identified by Gram staining and biochemical tests.

*Escherichia coli* O127:H6 E2348/69 (EPEC), *Salmonella enterica* serovar Typhimurium ATCC 14028, and *Staphylococcus aureus* ATCC 25923 were grown on Luria–Bertani (LB) agar at 37 °C for 24 h under aerobic conditions. Cultures were stored at −70 °C in Brucella broth (Becton, Dickinson and Company, Sparks, MD, USA) supplemented with 10% fetal bovine serum (Gibco BRL Life Technologies Corporation, Grand Island, NY, USA) and 50% glycerol. The strains were identified by Gram staining and biochemical tests.

### 4.3. Antibacterial Activity

All strains were evaluated for antimicrobial susceptibility according to Clinical and Laboratory Standards Institute [45] using the broth dilution method. Bacteria were grown in Mueller-Hinton broth (DIFCO, Becton Dickinson and Company, Sparks, MD, USA). For *H. pylori*, it was supplemented with 0.2% β-cyclodextrin, vancomycin (10 mg/L), trimethoprim (5 mg/L), amphotericin B (2 mg/L), and polymyxin B (2.5 mg/L). Different concentrations of the compounds (3.9–62.5 µg/mL), dissolved in dimethyl sulfoxide (DMSO), were added to the bacterial cultures at the beginning of the log growth phase (1 × 10^8^ CFU/mL). ΔA_600_ was measured after 24 h of incubation under microaerophilic conditions, 37 °C, and shaking at 150 rpm for *H. pylori*, and after 3.5 h of incubation at 37 °C, aerobic conditions, and 180 rpm for *E. coli*, *S. aureus*, and *S. enterica*; in all cases, the time corresponded to the end of the log phase of growth. Percentage inhibition was calculated relative to a culture growing only in the presence of solvent DMSO (100% growth). The final solvent concentration in the assay did not affects the experiments. The Minimum Inhibitory Growth Concentration (MIC) was defined as the lowest concentration of the compound at which there is no growth. Metronidazole and clarithromycin were used as positive controls for *H. pylori*, and ampicillin for the other bacteria.

### 4.4. Urease Assay

#### 4.4.1. Partial Purification of Urease

Urease was obtained from an *H. pylori* broth culture in the logarithmic phase of growth. Bacteria were washed twice with PBS pH 7.4 and resuspended in PBS/protease inhibitor cocktail (cOmplete Roche TM, Roche Applied Science, Mannheim, Germany). Subsequently, the sample was subjected to sonication at 40 W in a Branson 250 Sonicator (Branson Ultrasonics Co., Danbury, CT, USA) at 4 °C. This process was repeated for three intervals of 30 s, with a 30 s rest period between each cycle. The lysate was centrifuged at 13,500 rpm (Centronix Microcentrifuge, Spintron Inc. Metuchen, NJ, USA) for 10 min at 4 °C. The supernatant was used as the urease source and was stored at 4 °C until use. Protein content was quantified by Bradford’s method using bovine serum albumin as a standard [46].

#### 4.4.2. Urease Inhibition Assay

The urease activity was evaluated by quantifying the NH_4_^+^ liberated after the hydrolysis of urea using the colorimetric Berthelot method with some modifications [47]. The reaction included in a final volume of 130 µL, 3 µg protein of urease which were preincubated for 5 min at 37 °C with 5 µL of the compounds dissolved in DMSO at different concentrations (3.9–125 µg/mL), to start the reaction, 20 µL of 37.5 mM urea (5 mM final concentration) were added and incubated for 10 min at 37 °C. For the colorimetric reaction, 50 µL of 354 mM phenol and 100 µL of 345 mM NaOH/176 mM NaClO were added. After 5 min, the absorbance was recorded at 600 nm using a Bio-Rad 2550 EIA reader (Bio-Rad Laboratories, Inc., Pleasanton, CA, USA). Ammonium sulfate was used as a standard for the calibration curve. The percentage of enzymatic activity was calculated with respect to the activity without inhibitor. Acetohydroxamic acid (AHA) was used as a positive control.

### 4.5. DNA Gyrase Supercoiling Assay

The *E. coli* DNA Gyrase Drug Screening kit from TopoGEN (TopoGEN, Inc. Port Orange, FL, USA) was used. The kit is based on gyrase supercoiling activity on a relaxed plasmid DNA substrate. The reaction medium contained 4 µL of 5× buffer (35 mM Tris-Cl, pH 7.5, 24 mM KCl, 4 mM MgCl_2_, 2 mM dithiothreitol, 1.8 mM spermidine, 1 mM ATP, 6.5% glycerol, and 0.1 mg BSA/mL), 0.3 µL of relaxed plasmid pHOT1 (0.3 µg), and 0.15 µL of DNA gyrase (1 U/µL) in a final volume of 20 μL. To evaluate the effect of the compounds on DNA gyrase supercoiling activity, 1 µL of different concentrations (0.1–50 µM) of the compounds dissolved in DMSO was added before DNA gyrase. The reactions were incubated at 37 °C for 1.5 h. The products of the reactions were run on a 1% agarose gel at 50 V, stained with 0.5 µg/mL ethidium bromide, and photodocumented on a UV transilluminator. The supercoiled plasmid was quantified with ImageJ^®^ free software, version 1.54d. 100% of the activity corresponded to a reaction in which only the solvent (DMSO) was added. Ciprofloxacin (3 µM, final concentration) was used as a positive control.

### 4.6. In Vivo Gastroprotective Activity

#### 4.6.1. Animals

Adult male CD1 mice weighing 35–40 g provided by the vivarium of the Faculty of Medicine of UNAM were used. The animals were maintained at a controlled temperature of 21 ± 2 °C in 12/12 h light/dark cycles with free access to water and standard rodent food. The experimental protocol was approved by the Ethics Committee for the Care and Use of Laboratory Animals, Faculty of Medicine, UNAM, Mexico City, Mexico (CICUAL 076/2021) and was carried out in accordance with the Official Mexican Standard of Care and Animal Handling and in compliance with international guidelines for ethical standards for animal research.

#### 4.6.2. Anti-Ulcerogenic Activity

Acute gastric ulceration with ethanol was performed according to the method described by Bucciarelli [36]. The mice were divided into groups, *n* = 6. The negative control groups received either isotonic sodium saline solution (NSS) or the vehicle, 0.5% sodium carboxymethylcellulose (CMC), both at 7 mL/kg. Carbenoxolone, 50 mg/kg body weight (BW) in NSS, in the case of the positive control. The experimental groups received the compounds suspended in the vehicle at doses of 50, 100, and 200 mg/kg BW. All the samples were prepared freshly and administered by gastric gavage. After an hour of administration, all groups received orally absolute ethanol (7 mL/kg) to induce ulceration, and 1.5 h later, the mice were sacrificed in a CO_2_ chamber. Each stomach was dissected out, insufflated with 10% formalin, and fixed in the same solution for 24 h. The stomachs were opened along the greater curvature, pressed between two glass plates, and scanned. Areas of mucosal damage of the glandular stomach estimated in square millimeters were quantified with ImageJ^®^ free software. The ulcer index was calculated by the following expression:

(1) $$\mathrm{UI}=\frac{\mathrm{Ulcerated}\;\mathrm{area}}{\mathrm{Total}\;\mathrm{stomach}\;\mathrm{area}}$$

And the percentage of gastroprotection by:

(2) $$\%G=\frac{\mathrm{UI}\;\mathrm{control}-\mathrm{UI}\;\mathrm{test}}{\mathrm{UI}\;\mathrm{control}}\times 100$$

For histological analysis, a 10% formalin fixed glandular portion of the stomach was embedded in paraffin wax, and sagittal sections of 5 µm of thickness were obtained. Tissue sections were stained with hematoxylin-eosin (H&E) and were examined under the light microscope for either morphological or cellular changes.

### 4.7. Subacute Toxicity Assay

#### 4.7.1. Animals

CD1 male mice aged 6 to 7 weeks, obtained from the Centro de Investigación y de Estudios Avanzados del Instituto Politécnico Nacional (Cinvestav) animal facility, were used. Animals were randomly grouped in autoclaved bedding in microisolator cages (Super Mouse, model 750, Lab Products LLC, Aberdeen, MD, USA) within an OC 2100 mouse rack (Lab Products Inc., Seaford, DE, USA). Throughout the trial, they were maintained in pathogen-free conditions with 12/12 h light/dark cycles, at 20 and 24 °C, relative humidity 40–70%, and a noise level below 70 dB. The mice had free access to ultrafiltered tap water and sterilized chow (PicoLab Rodent Diet, 5058, Richmond, IN, USA). Animals were acclimatized for one week before experimentation. The experimental procedure was approved by Cinvestav’s Internal Committee for the Care and Use of Laboratory Animals (CICUAL, Protocol No. 0413-25) and complied with the Mexican Guideline Regulations of Animal Care and Maintenance and with international guidelines for ethical standards for animal research.

#### 4.7.2. Study Design

The subacute toxicity of DAC_2_-Cu and DAC_2_-Zn was assessed in CD1 mice according to Organisation for Economic Co-operation and Development (OECD) Test Guidelines 407 [44], with modifications. The animals were separated in three different groups (*n* = 5); the first was the control group (vehicle; 0.5% sodium carboxymethylcellulose (CMC) solution), the second and third groups received orally 200 mg/kg BW of DAC_2_-Cu or DAC_2_-Zn for a period of 28 days once a day. Three other groups (*n* = 4), corresponding to a recovery phase of the study, were included, and treated exactly as the previous ones, to observe the reversibility, persistence, or delayed onset of possible toxic effects. These groups were kept for 28 days without treatment after the last administration.

The animals were examined daily for signs of toxicity, such as changes in behavioral responses (locomotion, aggression), spontaneous activity (response to tail nibbling and noises), fecal appearance, tremors, bristling, and mortality. Their weight was monitored every third day. At the end of the experimental protocol, the mice were fasted for 12 h. Urine samples were collected during this period. The mice were then euthanized using isoflurane and dissected. Blood was drawn by cardiac puncture for the analysis of biochemical parameters, specifically for the determination of alanine aminotransferase (ALT) and lactate dehydrogenase (LDH), as well as for the determination of glucose, total protein, albumin, creatinine, and bilirubin. Biochemical parameters were determined using commercial Randox kits following the manufacturer‘s protocols (Randox Laboratories Ltd., London, UK). All organs, mucous membranes, body cavities, etc., were examined for macroscopic pathological alterations. Major organs (stomach, liver, kidney, heart, lung, and spleen) were weighed. Absolute values were recorded, and relative values (organ weight/body weight ratio) were calculated. The organs were preserved in 10% neutral buffered formalin. Histopathological examination of the liver and kidney was performed. Tissues were dehydrated, embedded in paraffin, and sectioned at 5 μm thickness. Sagittal sections were stained with H&E and examined under the microscope.

### 4.8. Statistical Analysis

The Kolmogorov–Smirnov test was used to determine the normal distribution of the data, comparisons by dose or concentration were analyzed with the nonparametric Kruskall-Wallis test, and Dunn’s post hoc test was used to determine significant differences between groups. For all tests, *p* < 0.05 was considered statistically significant. All data were reported as mean ± standard deviation (SD), except for gastroprotection results and body weight change analysis, which were reported as mean ± standard error of the mean (SEM). At least three independent experiments were performed in triplicate for each of the conditions. In animal tests, at least three animals were used per treatment. All statistical tests were performed using the R program, 4.2.1 version [48].

## 5. Conclusions

The current study highlights the anti-*H. pylori* properties of DAC and its four metal derivatives (DAC_2_-Cu, DAC_2_-Zn, DAC_2_-Mn, and DAC_2_-Mg). Interestingly, none of these compounds affected the growth of *E. coli*, *S. enterica*, and *S. aureus*, suggesting bacteriospecific activity against *H. pylori*. Moreover, DAC_2_-Zn and DAC_2_-Cu also inhibit *H. pylori* urease, with an IC_50_ lower than the positive control, AHA. Furthermore, these two compounds exhibit gastroprotective effects in mice, achieving approximately 70% protection at a dose of 200 mg/kg BW. It must be pointed out that despite administering this dose for 28 consecutive days to the animals, there was no mortality or irreversible organ damage, as confirmed by anatomical-histological examinations and clinical biochemical analysis performed on the animals.

Consequently, DAC_2_-Zn and DAC_2_-Cu show promise as novel alternatives for *H. pylori* eradication therapies and as antiulcer agents. Further studies are necessary before they can be implemented for human therapeutic applications.

## Figures and Tables

**Table 1:** Anti-*H pylori* activity of DAC and its metallic derivatives.

Compound	MIC ^1^	
	µg/mL	µM
Curcumin	1.95	5.29
DAC	62.50	138.12
DAC_2_-Cu	15.62	15.00
DAC_2_-Zn	31.25	29.86
DAC_2_-Mn	15.62	14.90
DAC_2_-Mg	31.25	28.84
Metronidazole ^2^	300.00	1752.84
Clarithromycin ^2^	0.05	0.07

^1^ MIC: Minimum inhibitory concentration. ^2^ Reference antibiotics for anti-*H. pylori* test.

**Table 2:** Growth inhibition of *E. coli*, *S. enterica* and *S. aureus* by DAC and its metallic derivatives.

Compound (62.5 µg/mL)	*E. coli* EPEC	*S. enterica*	*S. aureus*
	Inhibition (%)		
Curcumin (170 µM)	25.7 ± 3.3	47.5 ± 10.1	67.2 ± 4.0
DAC (138 µM)	21.3 ± 3.0	15.2 ± 2.4	16.7 ± 6.9
DAC_2_-Cu (60 µM)	10.7 ± 2.8	3.9 ± 1.2	10.9 ± 1.8
DAC_2_-Zn (60 µM)	19.3 ± 4.5	10.0 ± 3.1	23.7 ± 6.3
DAC_2_-Mn (56 µM)	8.6 ± 3.4	6.5 ± 4.1	10.9 ± 2.0
DAC_2_-Mg (58 µM)	14.2 ± 2.3	11.0 ± 8.7	17.4 ±1.7
Ampicillin (40 µM) (14 µg/mL)	100%		

**Table 3:** Percentage of gastroprotection.

Treatment	50 mg/kg	100 mg/kg	200 mg/kg
Carbenoxolone	78.3 ± 4.8		
Curcumin	-	65.5 ± 8.4	71.1 ± 3.4
DAC	24.8 ± 19.0	20.3 ± 13.4	18.6 ± 8.6
DAC2-Cu	19.0 ± 6.1	56.5 ± 7.0 *	71.2 ± 5.5 *
DAC2-Zn	43.6 ± 10.8	59.3 ± 6.5	76.6 ± 6.3
DAC2-Mn	20.8 ± 11.6	21.1 ± 16.5	24.0 ± 15.6
DAC2-Mg	0 ± 10.0	0.2 ± 19.5	11.4 ± 14.3

* Statistically significant differences with respect to the dose of 50 mg/kg of the compound (*p* < 0.05).

**Table 4:** Clinical biochemistry parameters on CD1 mice administered orally with 0.5% CMC (vehicle), DAC_2_-Cu (200 mg/kg BW), and DAC_2_-Zn (200 mg/kg BW) for 28 days.

	Experimental Phase			Recovery Phase		
Serum	Vehicle	DAC_2_-Cu	DAC_2_-Zn	Vehicle	DAC_2_-Cu	DAC_2_-Zn
Total protein	5.3 ± 0.3	5.5 ± 0.4	5.4 ± 0.4	5.2 ± 0.5	5.8 ± 1.0	5.6 ± 0.4
Albumin	3.5 ± 0.5	3.5 ± 0.4	3.5 ± 0.4	3.1 ± 1.01	3.4 ± 0.3	3.5 ± 0.7
Creatinine	0.37 ± 0.08	0.30 ± 0.06	0.34 ± 0.04	0.44 ± 0.18	0.37 ± 0.10	0.31 ± 0.14
Total bilirubin	0.17 ± 0.05	0.19 ± 0.062	0.16 ± 0.03	0.25 ± 0.04	0.34 ± 0.04	0.29 ± 0.05
Glucose	164.9 ± 52.8	178.7 ± 41.1	147.3 ± 14.0	200.6 ± 25.6	232.5 ± 29.2	203.6 ± 56.9
ALT	25.0 ± 14.8	12.3 ± 9.0	24.0 ± 8.2	21.5 ± 8.7	23.4 ± 12.0	20.0 ± 5.2
LDH	891.8 ± 395.8	1465.1 ± 636.7	1051.1 ± 220.2	1945.1 ± 1399.5	1789.7 ± 916.3	1205.8 ± 283.5
**Urine**						
Total protein	3.9 ± 0.2	3.8 ± 0.2	4.1 ± 0.2	5.1 ± 0.3	5.6 ± 0.06	5.4 ± 0.3
Creatinine	25.56 ± 1.9	22.17 ± 5.3	28.16 ± 6.9	16.33 ± 8.0	22.36 ± 4.9	25.89 ± 10.7
Glucose	0.7 ± 1.0	0.1 ± 0.5	1.5 ± 0.7	2.2 ± 2.2	3.4 ± 3.1	2.0 ± 1.6

ALT: Alanine aminotransferase. LDH: Lactate dehydrogenase. According to the nonparametric Kruskal–Wallis test, there are no statistical differences between the vehicle and the treatments (DAC_2_-Cu and DAC_2_-Zn) for any of the parameters (*p* < 0.05).

## Data Availability

The original contributions presented in this study are included in the article/Supplementary Materials. Further inquiries can be directed to the corresponding author.

## Supplementary Materials

The following supporting information can be downloaded at: https://www.mdpi.com/article/10.3390/molecules30193849/s1, Table S1: Effect of compounds (50 μM) on DNA gyrase supercoil activity; Figure S1: Body weight changes; Table S2: Frequency of there being no treatment-related findings in the appearance of vital mice organs; Figure S2: No treatment-related findings; Figure S3: Representative microphotographs of cells in degeneration/necrosis in the liver of DAC_2_-Cu and DAC_2_-Zn treated groups.

## Abbreviations

The following abbreviations are used in this manuscript:

DAC	Diacetylcurcumin
MIC	Minimum Inhibitory Concentration
PPI	Proton Pump Inhibitor
LD	Lethal Dose
DNA	Deoxyribonucleic acid
IC_50_	Half-maximal inhibitory concentration
AHA	Acetohydroxamic acid
CUR	Curcumin
SD	Standard Deviation
BW	Body weight
NSS	Isotonic sodium saline solution
VEH	Vehicle
CMC	carboxymethylcellulose
CAR	Carbenoxolone
SEM	Standard Error of the Mean
H&E	Hematoxylin & Eosin
ALT	Alanine aminotransferase
LDH	Lactate dehydrogenase
SDs	Solid dispersions
OECD	Organisation for Economic Co-operation and Development
UNAM	Universidad Nacional Autónoma de México
ATCC	American Type Culture Collection
LB	Luria–Bertani
CLSI	Clinical and Laboratory Standards Institute
DMSO	Dimethyl sulfoxide
CFU	Colony Forming Unit
CICUAL	Committee for the Care and Use of Laboratory Animals
