# Supplementary material for: Anti-Helicobacter pylori Activity and Gastroprotective Effects of Diacetylcurcumin and Four Metal Derivatives
Source: Molecules. 2025 Sep 23;30(19):3849. doi: 10.3390/molecules30193849 (PMC12525688; doi:10.3390/molecules30193849)
Supplement: Supplementary file 1 [file molecules-30-03849-s001.zip › molecules-3858107-supplementary.pdf]

Supplementary information

Content

1. *Table S1:* Effect of compounds (50 µM) on DNA gyrase supercoil activity.
2. *Figure S1:* Body weight changes.
3. *Table S2:* Frequency of no treatment-related findings in the appearance of vital mice organs. *Figure S2*: No treatment-related findings.
4. *Figure S3.* Representative microphotographs of cells in degeneration/ necrosis in the liver of DAC_2-Cu and DAC_2-Zn treated groups.

Table S1. Effect of curcumin, diacetylcurcumin (DAC), and their metal derivatives (50 µM) on DNA gyrase supercoiling activity.

**Compound**	**Inhibition (%)**
Curcumin	6.6 ± 1.4*
DAC	-22.4 ± 13.3*
DAC_2-Cu	-4.4 ± 9.6*
DAC_2-Mg	-14.6 ± 8.8*
DAC_2-Mn	40.1 ± 9.5
DAC_2-Zn	26.3 ± 3.1
Ciprofloxacin 3 µM	100

Negative inhibition percentages were considered to lack inhibitory activity. Values are reported as the mean of three experiments (*two experiments) ± SD.

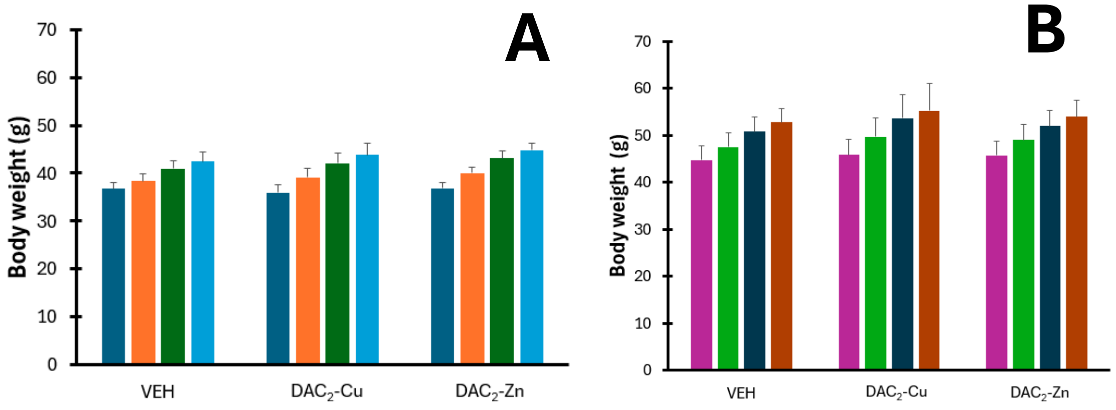

Figure S1. Body weight changes observed during subacute oral toxicity study. (A) Animals treated for 28 days (n=9), and (B) recovery phase groups (n=4). There were no significant differences ($p < 0.05$) in body weight between vehicle- and DAC_2-Cu and DAC_2-Zn-treated mice. The data is presented as mean ± SEM.

Table S2. Frequency of no treatment-related findings in the appearance of vital mice organs.

		**Frequency**				
	Finding	Lung		Liver		Stomach
**Phase**	Treatment	Darkening of the right upper lobe	General pallor	Slight yellowness appearance	Friability	Distension
**Experimental**	Vehicle	1/5	0/5	0/5	0/5	0/5
	DAC_2-Cu	1/5	0/5	0/5	0/5	0/5
	DAC_2-Zn	3/5	0/5	0/5	0/5	0/5
**Recovery**	Vehicle	1/4	2/4	1/4	0/4	0/4
	DAC_2-Cu	2/4	0/4	0/4	1/4	0/4
	DAC_2-Zn	1/4	0/4	1/4	1/4	1/4

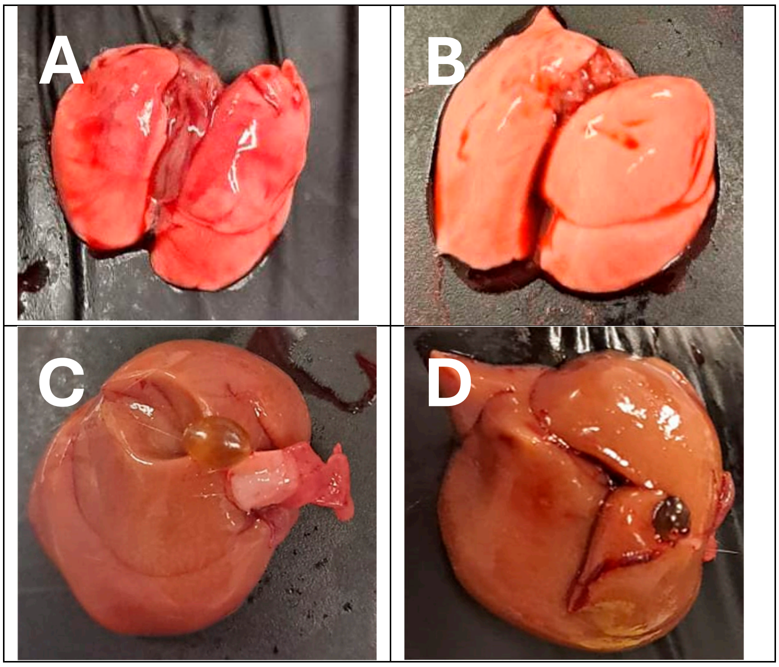

Figure S2. No treatment-related findings. (A) appearance of the normal lung. (B) pale lung. (C) appearance of the normal liver and (D) slight yellowness liver.

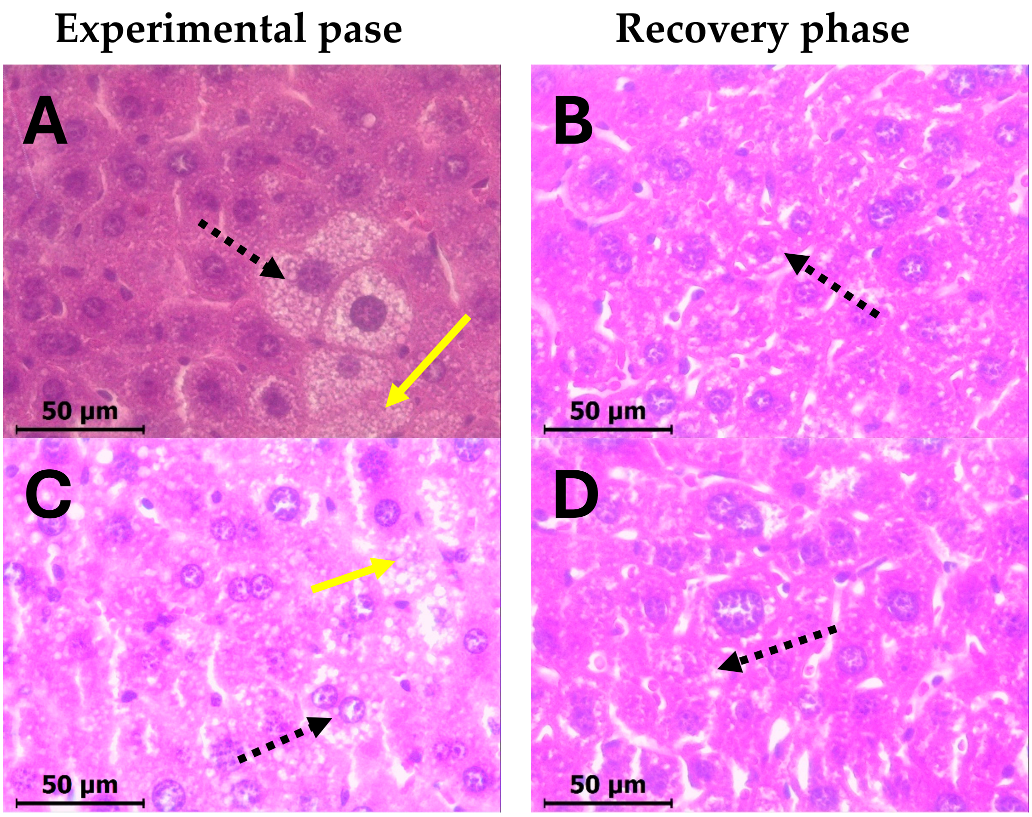

Figure S3. Representative microphotographs of mouse liver section after DAC_2-Cu and DAC_2-Zn administration. (H&E, 630x). (A and B) DAC_2-Cu, 200 mg/kg BW and (C and D) DAC_2-Zn, 200 mg/kg BW. A and C corresponds to the experimental phase and, B and D to the recovery phase. Vacuolar degeneration (dotted black arrows). Apoptotic cells (yellow arrows).
